# Intravenous injection of a modified vaccinia ankara virus induces intratumoral CD8+ lymphocyte infiltration favoring tumor control in an orthotopic tumor model upon combinatorial treatment with a TLR9 agonist

**DOI:** 10.1186/2051-1426-2-S3-P57

**Published:** 2014-11-06

**Authors:** Laetitia Fend, Tanja Gatard-Scheikl, Jacqueline Kintz, Murielle Gantzer, Emmanuelle Schaedler, Karola Rittner, Sandrine Cochin, Sylvie Fournel, Xavier Préville

**Affiliations:** 1 Transgene, France; 2 Transgene, Illkirch-Graffenstaden Cedex, France; 3 Transgene, Gambia; 4 CNRS-Université de Strasbourg, Illkirch Graffenstaden cedex, France

## Background

There are many critical steps for the clinical efficacy of antigen-specific T cell-based immunotherapies among which traffic to and access to tumor beds of effector T cells, are the most limiting [1]. Preclinical research most often uses ectopic mouse tumor models for the readout fastness and easiness provided by such models. However, those models may not be the optimal way to address the issue of targeting effector T cells to a deep organ born tumor.

## Methods

We set up an orthotopic preclinical model in which renal carcinoma cells (RenCa) expressing the human mucin 1 tumor-associated xeno-antigen are injected into the sub-capsular space of kidney in BALB/c mice. We then evaluated the impact of the route of injection on the therapeutic efficacy of a Modified Vaccinia virus Ankara expressing the human mucin 1 xeno-antigen (MVA-MUC1).

## Results

We show that intravenous administration of MVA-MUC1 is therapeutically superior to subcutaneous injection. Therapeutic efficacy of MVA-MUC1 can be further enhanced by intravenous administration of a TLR9 ligand (ODN1826). Substantial and necessary infiltration of tumor-bearing kidneys by CD8^+ ^lymphocytes is associated with therapeutic protection. We observe that infiltration of CD8^+ ^lymphocytes is not limited to tumor-bearing kidneys and is also detected in naive kidneys, liver and lungs. Kidney infiltrating lymphocytes are characterized by an effector memory phenotype and express PD-1 and Tim3 immune checkpoints molecules. Biodistribution experiments indicate that MVA encoded antigens quickly reach deep organs and in particular APC from the spleen upon i.v. injection of MVA in comparison to s.c. injection, resulting in a faster generation of MUC1 specific CD8^+ ^T cells. Unexpectedly, addition of ODN1826 to MVA-MUC1 immunization does not result in an increase in the frequency of MUC1 specific T cells detected in the spleen. Instead, ODN1826 was associated with a more pronounced modification of the tumor microenvironment towards a T_H_1 type inflammatory response and a recruitment of activated lymphocytes.

## Conclusions

Intravenous injection of MVA-MUC1 is associated with therapeutic efficacy and deep organ infiltration by MUC1-specific CD8^+ ^T cells in an orthotopic model of renal carcinoma. This therapeutic efficacy can be further increased through the use of ODN1826. This study supports the clinical evaluation of MVA-based immunotherapies via the intravenous route.
